# Medical YouTube Videos and Methods of Evaluation: Literature Review

**DOI:** 10.2196/mededu.8527

**Published:** 2018-02-12

**Authors:** Brandy Drozd, Emily Couvillon, Andrea Suarez

**Affiliations:** ^1^ McGovern Medical School of UTHealth Houston Houston, TX United States; ^2^ School of Medicine Georgetown University Washington, DC United States

**Keywords:** social media, YouTube, internet, health literacy, online education, videos

## Abstract

**Background:**

Online medical education has relevance to public health literacy and physician efficacy, yet it requires a certain standard of reliability. While the internet has the potential to be a viable medical education tool, the viewer must be able to discern which information is reliable.

**Objective:**

Our aim was to perform a literature review to determine and compare the various methods used when analyzing YouTube videos for patient education efficacy, information accuracy, and quality.

**Methods:**

In November 2016, a comprehensive search within PubMed and Embase resulted in 37 included studies.

**Results:**

The review revealed that each video evaluation study first established search terms, exclusion criteria, and methods to analyze the videos in a consistent manner. The majority of the evaluators devised a scoring system, but variations were innumerable within each study’s methods.

**Conclusions:**

In comparing the 37 studies, we found that overall, common steps were taken to evaluate the content. However, a concrete set of methods did not exist. This is notable since many patients turn to the internet for medical information yet lack the tools to evaluate the advice being given. There was, however, a common aim of discovering what health-related content the public is accessing, and how credible that material is.

## Introduction

In today’s world, the internet and social media are a part of everyday life. Within seconds, a handheld device can provide more information than one can possibly read. The ease and simplicity of finding information on the internet translates directly to answering health questions and concerns. By 2011, 59% of adults were looking up health information online, and internet access has expanded exponentially since then [1]. One of the most frequently used social media sites is YouTube, which was created in 2005 and now has over one billion users, allowing for hundreds of millions of hours of total video watch time each day [2]. Social media has great potential to provide easy access to medical information, but it is likely that the information received is neither accurate nor free of bias. A YouTube search on tanning bed use gives results with 68% of the videos having a positive view of bed use, with no mention of dangers such as melanoma. This is an obvious problem for the field of dermatology to address [3]. Issues related to online videos for patient education and their quality and accuracy have drawn more attention recently. Analyses of YouTube videos on heart failure, mammography, and asthma among others have been published since 2015, but there are no standardized methods or guidelines of evaluation [4-7]. The lack of regulation within online medical education is hindering progress made by physicians, but with knowledge of how YouTube videos can be assessed, the public as well as health care providers can better assess the quality of information they are receiving. The goal of this review is to determine how studies have been able to evaluate educational videos and to give an overall look at the most common methods used.

## Methods

A thorough search was performed within both Embase and PubMed in November 2016. A data management librarian determined the search terms after a preliminary search to find which key words would supply relevant articles. Many search combinations did not generate any articles as this is a relatively new topic and YouTube was not created until 2005. Thus, our inclusion date for articles encompassed anything published after the year 2005. PubMed and Embase were chosen as the literature databases to search, as they are reputable sources of medical literature and PubMed also includes literature from the Medline database. The first search was performed in Embase with the term “patient education” AND “YouTube” OR “Online Videos” OR “Online video.” In PubMed, two separate searches were performed. The first search term was (“Patient Education as Topic” [Medical Subject Headings] OR “patient education”) AND (“YouTube” OR “online videos”), and the second search term was “YouTube health guidelines.” One author analyzed all of the included articles, and the results were reviewed and approved by another author. Each included article was read in its entirety, and the methods as well as unique characteristics for each study were recorded in MS Excel formatting and compared.

The inclusion criteria for the studies to be reviewed were as follows: (1) analysis of videos intended for patients or guardians, (2) contains detailed and repeatable methods of analysis, (3) English language, and (4) analysis of videos that are made available to the public.

**Figure 1:**
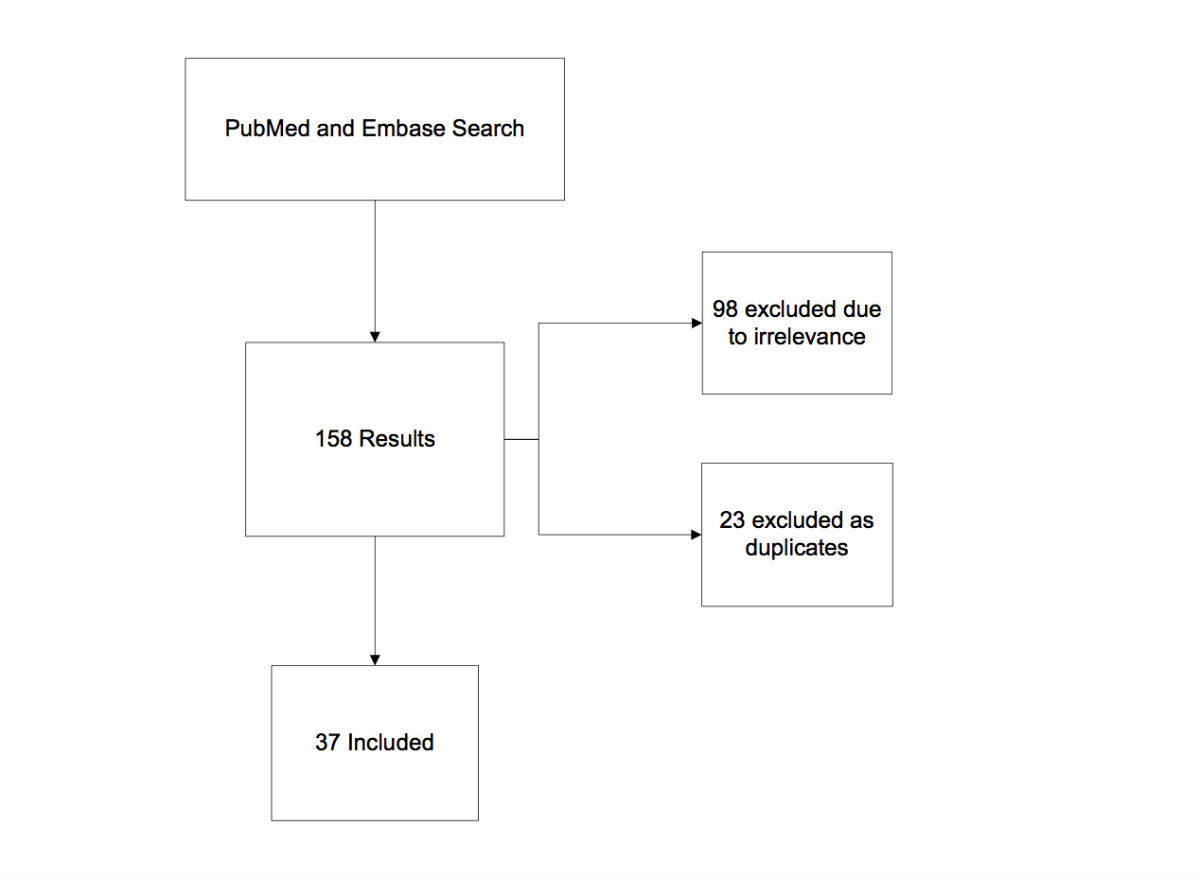
Search results and excluded studies.

The Embase search (“patient education” AND “YouTube” OR “Online Videos” OR “Online video”) generated 65 results, of which 20 were included for review. The first PubMed search (“Patient Education as Topic” [Medical Subject Headings] OR “patient education”) AND (“YouTube” OR “online videos”) had 77 results and 13 articles met the inclusion criteria. The last PubMed search (“YouTube health guidelines”) gave 16 results, of which 4 articles were reviewed. This resulted in a total of 37 studies to be reviewed (see Multimedia Appendix 1). Most excluded articles were left out due to irrelevance, meaning that the studies focused more on websites than videos, tested the efficacy of personal physician-created videos on their own patients, or the videos analyzed were intended for physician use only. The excluded studies are summarized in Figure 1.

This study was exempt from requiring Institutional Review Board approval.

## Results

The same chronological process was generally followed within each piece of literature reviewed, but no two video evaluations were performed in an identical manner. The first step within each study included determining the search term(s) to be used. Multiple search terms were used to ensure that all possible patient searches could be evaluated. For example, “gallbladder disease,” “gallstone disease,” and “gallstone treatment” were all used to assess YouTube videos about gallstone disease, as they are likely terms used by the public [7]. Other studies used search operators in order to create comprehensive search terms [8]. Another method used for searching techniques was multiple search dates.

Methods and techniques used to determine search terms and searching criteria for YouTube videos included multiple search terms (20 studies), autocomplete function within search bar, use of search operators, multiple search dates, limited number of pages within a particular search to be analyzed (30 studies), and changing the video results to be sorted by “most viewed” (3 studies).

The next step involves determining which videos should be included in the study. Some researchers set a maximum time limit for included videos. Only one study excluded videos based on number of views, in which the videos were required to have greater than 2500 views [6]. The predetermined inclusion criteria used by various studies were English language; must not be a duplicate video; must have audio; videos directed towards the public and not only a physician; video length not greater than a predetermined maximum number of minutes (7 studies), most commonly being 10 minutes (4 studies); and must have a predetermined number of video views (1 study).

Most studies had multiple reviewers and stated the qualifications of the reviewers, which included students, residents, or physicians. The most rigorous qualification requirements involved a 1-month clinical rotation in the department of allergy and clinical immunology and successful completion of a series of learning objectives [5]. The videos were reviewed separately, followed by comparison of results, but how the differences were settled varied. The most common method deferred the discrepancy to another qualified individual or physician who would determine the final result. One study averaged the individual reviewer scores and accepted that result as the final evaluation [9]. Since these evaluations are largely subjective, interrater reliability was assessed in 15 of the articles through the calculation of a kappa score.

The source of upload allowed for categorization of videos. An analysis of educational videos on children’s dental caries separated the videos into health care professionals, academic institutes, professional organizations, individual users, and product companies [10]. These were the most common source categories, but others included news agencies and health care websites as well. Three of the studies assessed the reliability of the upload sources through a modified DISCERN method for which the reliability score ranges from 0 to 5. The criterion from the original DISCERN model were clear aims, balanced/unbiased, reliable sources of information, additional resources provided, and mention of uncertainty [11-13].

To determine the accuracy of the videos, 22 of the studies created a novel scoring system. These scoring systems and other methods are summarized in Table 1. In a study on the accuracy of YouTube videos about stopping epistaxis, a point was awarded for each of the necessary steps mentioned [14]. In another study, the scoring ranged from -10 to 30, where a point was awarded for each accurate piece of information included, and a point was subtracted for each incorrect fact that could harm a patient [5]. Through the *Journal of the American Medical Association* guidelines used, a point is given for authorship, disclosure, source, and currency of the video [15]. Health on the Net (HON) Foundation has also created a set of 8 principles for websites to abide by called the HONcode [16]. Another method of evaluation was categorization of videos as useful, misleading, or as personal experiences. A useful video contains accurate information about any facet of the disease such as epidemiology, treatments, and procedures performed, and is misleading if it presents inaccurate information or promotes a scientifically unproven treatment [17].

Ten studies evaluated the quality of the video presentation, of which five assessed video quality according to global quality score guidelines. This rates the quality from a score of 1-5 while taking into account video flow and usefulness [11,18]. Other quality assessment guidelines constructed by reviewers included evaluation of lighting, audio, and number of pixels as well as other video characteristics [7,19].

**Figure 2:**
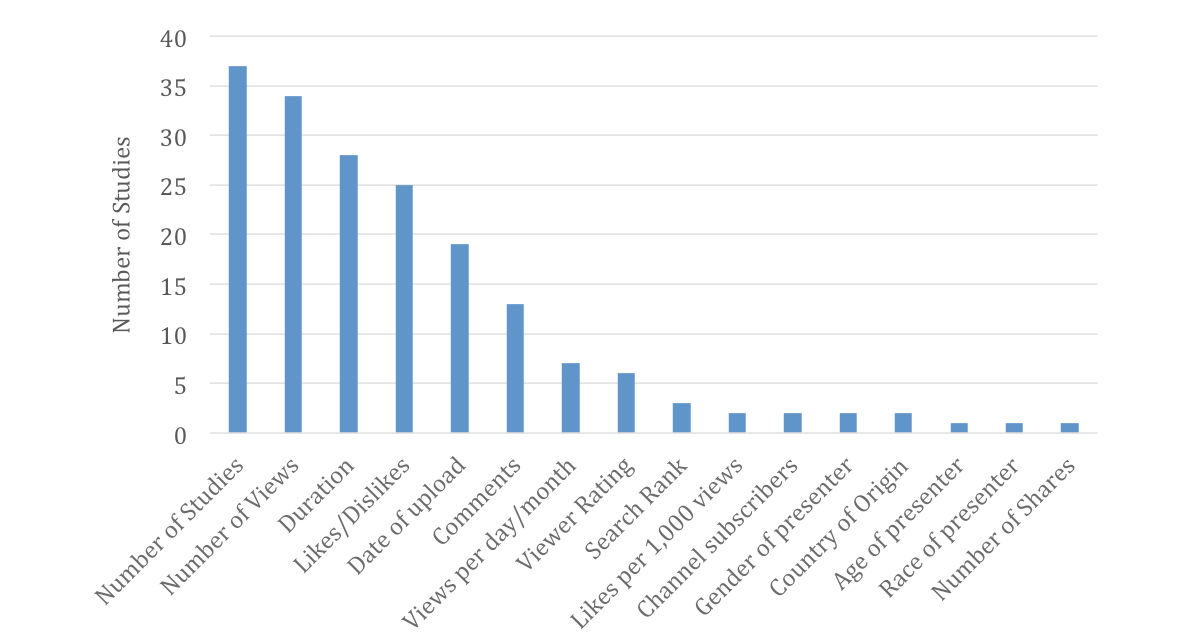
Video data collected by various studies as of Nov 2016 (results based on all 37 studies reviewed).

The most common video characteristics recorded were number of views, followed by source of upload. These data, along with the frequencies of other parameters taken into account by the various studies, are summarized in Figure 2. In addition, 5 studies measured popularity by either calculating likes per 1000 views or views per day/per month.

The most common sequence of methods performed is as follows: (1) determine a search term(s); (2) establish inclusion criteria for videos; (3) determine video reliability scoring/what parameters will be taken into account; (4) review videos individually; (5) convene to discuss discrepancies and determine final results; and (6) analyze results and determine the reliability or usefulness of videos and which characteristics determine that quality.

**Table 1:** Methods for determining accuracy and usefulness of videos.

Method	Description
Creation of a novel scoring system (22 studies)	Formation of guidelines based on scientific literature and physician expertise with a corresponding point system
HONcode^a^	Health on the Net Foundation guidelines for websites adapted for YouTube videos
*Journal of the American Medical Association* website guidelines	Adaptation of these guidelines to be implemented for YouTube videos
Judgment as useful, misleading, or personal experiences	Subjective categorization by the researchers based on knowledge of the topic as well as on predetermined criteria

^a^Health on the Net (HON) Foundation created a set of 8 principles for websites to abide by called the HONcode.

## Discussion

### Principal Findings

Our review found that defining a search term, determining how to judge or score the videos, and determining the reliability of the video sources and information were the primary methods discussed throughout the studies. There were also many steps taken to ensure that the evaluations were indicative of how the general public and patients would receive and understand the information given in the YouTube videos. For example, YouTube content is constantly being modified, thus researchers performed content searches at later dates to give insight into the evolution of viewership [18]. They also took measures to include search terms that were more likely to be used by patients. A recent study on consumer health-related activities on social media determined that much of the involvement was based on convenience [20]; many researchers limited the page number and search rank of the videos to be included in their analysis. Most people searching YouTube do not take the time to look at search results in later pages, and evaluating these videos would not be an accurate representation of what the public is viewing. The previous study on consumer health-related social media activities also revealed that many social media users turn to the internet for emotional support during a chronic disease or illness [20]. Within the studies of this literature review that judged videos as either useful, misleading, or personal experiences, there was an emphasis on the personal experience videos, which were further evaluated for accuracy. This was not without reason as many patients feel that the information provided by their physicians is not sufficient and they turn to their online peers for support [20].

Throughout the reviewed literature, there was considerable focus on determining which video characteristics could be quantified and compared to reveal a positive correlation with video accuracy. The most commonly statistically analyzed parameters were video score versus number of likes, and video score versus source of upload. One study discovered that younger patients as well as patients with higher education are more likely to use the internet as source of health information due to their increased ability to search the Web and identify reliable information and sources [21]. If video parameters and sources can be linked to predictability of accuracy, then perhaps patients within the health literacy gap will feel more confident in navigating this pool of easily accessible medical knowledge.

### Limitations

This paper is a comprehensive review, but it is not a systematic review. All efforts were taken to include all articles possible, but we cannot guarantee that some were not missed. In addition, this is a newly popular topic and it is likely that use of these search terms at a later date will result in an increased number of results.

### Conclusions

Social media has the potential to aid in closing the health literacy gap and can present information in novel ways that allow even illiterate populations to learn [22]. The Internet has increased opportunities for open discussion about health and medicine as well as a created a platform for moral support [22]. However, with this increased opportunity also comes a chance for dissemination of inaccurate and even harmful information. Physicians and researchers have realized the increased impact of social media on the knowledge and compliance of their patients, as evidenced by a recent increase in published studies regarding medical YouTube video reliability. Thus, these general steps as well as the unique processes detailed throughout this review could be of use to patients in search of online medical advice. While a common sequence of methods was able to be determined, there are no substantial similarities between study methods. The inconsistency stems from the fact that there are a multitude of possible variables that contribute to both the popularity and the efficacy of educational videos. This creates a barrier to analysis duplication and the formation of a systematic process that ensures adequate information and regulation for patients. However, there was a common sequence of steps found. This topic will be an ongoing field for further research as social media engagement continues to increase across the world and as more people realize the dire need for increased health education in all populations.

## Appendix

Multimedia Appendix 1 Titles of the included studies that complied with all inclusion criteria.

## Abbreviations

HON: Health on the Net
