# Supplementary material for: Medical YouTube Videos and Methods of Evaluation: Literature Review
Source: JMIR Med Educ. 2018 Feb 12;4(1):e3. doi: 10.2196/mededu.8527 (PMC5826977; doi:10.2196/mededu.8527)
Supplement: Multimedia Appendix 1 [file mededu_v4i1e3_app1.pdf]

Multimedia Appendix 1. Titles of the included studies.

YouTube as a Source for Parents' Education on Early Childhood Caries
Evaluation of educational content of YouTube videos relating to neurogenic bladder and intermittent catheterization.
The Validity of YouTube Videos on Pediatric BLS and CPR.
Are YouTube videos accurate and reliable on basic life support and cardiopulmonary resuscitation?
YouTube as a Source of Information on Tanning Bed Use
YouTube as a Source of Information for Children with Paroxysmal Episodes
YouTube as a source of information about retinitis pigmentosa
YouTube as a source of patient information on gallstone disease
YouTube As an Information Source for Femoroacetabular Impingement: A Systematic Review of Video Content
YouTube as a source of information on dialysis: a content analysis
Benign prostatic hyperplasia related content on YouTube: unregulated and concerning
YouTube as a source of useful information on diabetes foot care
YouTube videos as a source of medical information during the Ebola hemorrhagic fever epidemic
Analysis of YouTube as a source of information for peripheral neuropathy
Assessment of YouTube videos as a source of information on medication use in pregnancy
Online Curves: A Quality Analysis of Scoliosis Videos on YouTube
Social media patient testimonials in implant dentistry: information or misinformation?
Heart Failure Videos on YouTube - the Good, the Bad, and the Ugly: A Study on the Utility and Education Value for Patients, Health care Practitioners and Learners
YouTube as Source of Prostate Cancer Information
YouTube as a Source of Information on Kidney Stone Disease
YouTube Videos as a Teaching Tool and Patient Resource for Infantile Spasms
YouTube for Information on Rheumatoid Arthritis −− A Wakeup Call?
YouTube® and inflammatory bowel disease
YouTube: A good source of information on pediatric tonsillectomy?
Patient information on breast reconstruction in the era of the world wide web. A snapshot analysis of information available on youtube.com
YouTube as an information source for pediatric adenotonsillectomy and ear tube surgery
Utilization of YouTube as a tool to assess patient perception regarding implanted cardiac devices
YouTube as a source of chronic obstructive pulmonary disease patient education
YouTube™ as a source of patient information for lumbar discectomy
Quality of YouTube videos for patient education on how to use asthma inhalers
Can We Rely on Internet in the Era of Hepatitis C Cure?
Popular on YouTube: A critical appraisal of the educational quality of information regarding asthma
Characteristics of YouTube™ Videos Related to Mammography
Assessing the Content of YouTube Videos in Educating Patients Regarding Common Imaging Examinations
A qualitative analysis of methotrexate self-injection education videos on YouTube
How to stop a nosebleed': An assessment of the quality of epistaxis treatment advice on YouTube
YouTube provides irrelevant information for the diagnosis and treatment of hip arthritis
